# Preventable Hospitalizations in Adults With Alzheimer's Disease and Related Dementias: United States, 2016–2018

**DOI:** 10.1093/geroni/igab046.1183

**Published:** 2021-12-17

**Authors:** Benjamin Olivari, Christopher Taylor, Roshni Patel, Raza Lamb, Matthew Baumgart, Lisa McGuire

**Affiliations:** 1 Centers for Disease Control and Prevention (CDC), Atlanta, Georgia, United States; 2 Center for Disease Control and Prevention, Atlanta, Georgia, United States; 3 Alzheimer's Association, Chicago, Illinois, United States; 4 CDC, Atlanta, Georgia, United States

## Abstract

Alzheimer's disease and related dementias (ADRD) are a significant public health burden. Preventing hospitalizations in adults with ADRD is a public health priority. Data from the 2016–2018 Healthcare Cost Utilization Project National Inpatient Sample, an all-payer representative sample of US hospitalizations, were used to describe potentially preventable hospitalizations in adults ≥45 years with ADRD using International Classification of Disease, Tenth Edition, Clinical Modification (ICD-10-CM) codes. Definitions for principal or any-listed ICD-10-CM codes from the Agency for Healthcare Research and Quality defined potentially preventable hospitalizations where admissions might have been avoided by appropriate outpatient primary care management. Of discharges in adults ≥45 years with a potentially preventable hospitalization diagnosis, 11.4% (N=389,155) had a diagnosis of ADRD listed in any position. Of those discharges with ADRD, a significantly higher proportion (82.6%) with diagnosis related to potentially preventable hospitalizations were aged ≥75 years compared to 78.9% without potentially preventable hospitalizations. Additionally, of those with ADRD and potentially preventable hospitalization diagnoses, a higher proportion died in the hospital (5.7%) compared to those without potentially preventable hospitalization diagnoses (3.4%). The most common potentially preventable hospitalization diagnoses among adults with ADRD were related to sepsis (34.0%), injuries (20.8%), urinary tract infections (14.2%), and heart failure (12.7%). Measures focusing on preventing injuries as well as identifying early signs and symptoms of potentially preventable hospitalizations like urinary tract infections and sepsis in adults with ADRD could reduce the number of preventable hospitalizations in this population.

